# Leptospirosis in “Eco-Challenge” Athletes, Malaysian Borneo, 2000

**DOI:** 10.3201/eid0906.020751

**Published:** 2003-06

**Authors:** James Sejvar, Elizabeth Bancroft, Kevin Winthrop, Julie Bettinger, Mary Bajani, Sandra Bragg, Kathleen Shutt, Robyn Kaiser, Nina Marano, Tanja Popovic, Jordan Tappero, David Ashford, Laurene Mascola, Duc Vugia, Bradley Perkins, Nancy Rosenstein, the Eco-Challenge Investigation Team

**Affiliations:** * Centers for Disease Control and Prevention, Atlanta, Georgia, USA; † California Department of Health Services, Berkeley, California, USA; ‡ Los Angeles County Department of Health Services, Los Angeles, California, USA

**Keywords:** leptospirosis, febrile illness, adventure travel immunoassay, doxycycline, research

## Abstract

Adventure travel is becoming more popular, increasing the likelihood of contact with unusual pathogens. We investigated an outbreak of leptospirosis in “Eco-Challenge” multisport race athletes to determine illness etiology and implement public health measures. Of 304 athletes, we contacted 189 (62%) from the United States and 26 other countries. Eighty (42%) athletes met our case definition. Twenty-nine (36%) case-patients were hospitalized; none died. Logistic regression showed swimming in the Segama River (relative risk [RR]=2.0; 95% confidence interval [CI]=1.3 to 3.1) to be an independent risk factor. Twenty-six (68%) of 38 case-patients tested positive for leptospiral antibodies. Taking doxycycline before or during the race was protective (RR=0.4, 95% CI=0.2 to 1.2) for the 20 athletes who reported using it. Increased adventure travel may lead to more frequent exposure to leptospires, and preexposure chemoprophylaxis for leptospirosis (200 mg oral doxycycline/week) may decrease illness risk. Efforts are needed to inform adventure travel participants of unique infections such as leptospirosis.

Each year, 60 million Americans travel abroad. Increasingly, these persons are traveling to more remote and exotic destinations. Adventure travel is now the largest growing segment of the leisure travel industry, with a growth rate of 10% per year since 1985 (Adventure Travel Society, pers. comm.). This travel has led to an increasing risk for contact with pathogens uncommon in industrialized countries, especially for participants in adventure sports and extreme travel. Both of these pursuits may predispose persons to infection with unusual organisms through exposures to lakes, rivers, caves, and canyons, as well as insect vectors. These illnesses may be unfamiliar to practitioners in the travelers’ home countries, and symptoms may go unrecognized. Leptospirosis, a bacterial zoonotic infection, is more frequently found in developing countries, and its protean early symptoms may be difficult to diagnose clinically. Prevention of leptospirosis in humans has previously relied on mechanical barriers and avoidance (*1*), but limited published data suggest that preexposure chemoprophylaxis may be beneficial to some groups (*2*–*4*).

From September 7 to September 11, 2000, the Idaho Department of Health, the Los Angeles County Department of Health Services, and the GeoSentinel Network (an international surveillance network of travel clinics) notified the Centers for Disease Control and Prevention (CDC) of at least 20 cases of febrile illness. The illness was characterized by the acute onset of high fever, chills, headache, and myalgias; major laboratory test abnormalities and important pulmonary or central nervous system involvement were absent. All ill persons had participated in the Eco-Challenge-Sabah 2000 multisport endurance race, held in Malaysian Borneo August 21–September 1, 2000. Three hundred four athletes from 26 countries and 29 U.S. states competed in the 10-day endurance event. Segments of the event included jungle trekking, prolonged swimming and kayaking (both in fresh and ocean water), spelunking (caving), climbing, and mountain biking. Symptoms and exposure history, as well as initial laboratory testing, suggested that the illness was leptospirosis. We undertook an investigation to determine the etiology of the illness in the athletes and to make public health recommendations. We report on the results of this investigation and discuss recommendations for preventing leptospirosis in adventure travelers.

## Methods

### Epidemiologic Investigation

Upon identification of the first ill athletes, a complete list of U.S. and international participants in the Eco-Challenge event, including their telephone numbers and email addresses, was obtained from the race organizers. In addition, race organizers and some athletes were interviewed to determine details of the race activities, geography of the course, and possible exposures. Athletes in the United States were contacted by telephone, either by CDC or by representatives of the athletes’ state or local health departments, between September 7 and October 30, 2000. International athletes were contacted by representatives from their local ministries of health after notification by local GeoSentinel sites or the World Health Organization.

A standardized telephone questionnaire was administered, directed at determining demographics, symptoms, duration of illness, previous antibiotic use, and various exposures encountered during the event. A clinical case of illness was defined as onset of self-reported fever between August 21 and September 30, 2000, in an Eco-Challenge athlete, along with two or more of the following symptoms: chills, myalgias, headache, diarrhea, or conjunctivitis. We compared clinical case-patients with controls from the cohort of athletes to identify risk factors for illness.

### Laboratory Investigations

Thirty-eight serum samples were obtained from a convenience sample of the cohort (who met the clinical case definition) for laboratory testing for various pathogens. All submitted samples were tested for *Leptospira*-specific immunoglobulin (Ig) M antibodies by dot-enzyme-linked immunosorbent assay (ELISA) dipstick (Dip-S-Ticks immunoassay, Integrated Diagnostics, Baltimore, MD) or by microplate IgM-ELISA (PanBio Ltd., Brisbane, Australia), according to the manufacturer’s instructions. Laboratory evidence for leptospirosis was defined as a positive result for *Leptospira*-specific IgM antibodies.

All 38 samples were subsequently tested by microscopic agglutination test (MAT) by using a standard method (*5*), with 23 live antigen suspensions representing 17 serogroups. A titer of >200 against any of the antigens was considered positive evidence for a probable case of leptospirosis.

Given the broad differential diagnosis for febrile illness in the tropics, 18 submitted samples were also tested for alternative organisms. Samples with antibody responses to *Orientia tsutsugamushi*, *Rickettsia prowazekii,* and *R. typhi* were assayed by using a variation of the standard microimmunofluorescence test for rickettsiae (*6*). Testing for flaviviruses (dengue, Japanese encephalitis, Powassan/tick-borne encephalitis), alphaviruses (Chikungunya), bunyaviruses (Snowshoe Hare virus), and hantaviruses (Sin Nombre) was performed with the standard IgM ELISA assay (*7*).

### Statistical Analysis

Results of the telephone survey were entered into EpiInfo v. 6.04b (CDC, Atlanta, GA); the dataset was then imported into SAS v. 6.12 and v. 8.2 (SAS Institute Inc., Cary, NC) for subsequent analysis. Because laboratory testing was only available from a subset of the cohort, athletes were categorized on the basis of the clinical case definition. All variables were examined using PROC GENMOD (SAS). Those factors significant by univariate analysis, as well as known risk factors, were examined in a multivariable logistic regression model. Colinearity and interaction among variables were calculated by using standard statistical techniques.

## Results

### Epidemiologic Investigation

Of the 304 athletes competing in the Eco-Challenge event, 189 (62%) were contacted, including 129 (92%) of the 140 U.S.-based athletes. The median age of the contacted cohort was 34 years (range 21–50 years); 94% were white, and 74% were men. No significant differences were found between ill athletes who met the case definition and non-ill athletes by age, race, or gender. The most common symptoms athletes reported included fever, chills, muscle aches, headache, and diarrhea (Table 1). Conjunctival suffusion, a hallmark finding in persons infected with leptospires, was reported by 40 (21%) athletes. Joint aches and calf/leg pain were also frequently reported. The status of athletes with respect to residence in an area endemic for leptospirosis was unknown.

**Table 1:** Self-reported clinical symptoms in Eco-Challenge athletes

Symptom	% (n=189)
Chills	50
Muscle aches	50
Fever	49
Headache	47
Diarrhea	33
Joint aches	30
Calf/leg pain	30
Red eyes	22

Of the 189 athletes contacted, 80 (42%) met the clinical case definition. The median interval between the start of the race and onset of fever was 15 days (range 1–24 days), with a peak onset of fever on September 4, 2000 (Figure); no cases were detected after September 13. The median duration of illness was 7 days (range 1–17 days); 29 (36%) of the athletes who met the clinical case definition were hospitalized. No deaths were reported. Jaundice, pulmonary hemorrhage, meningoencephalitis, or other severe manifestations of leptospirosis were not reported.

**Figure:**
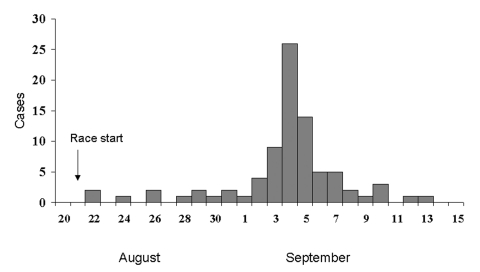
Date of fever onset for suspected and confirmed cases of leptospirosis in Eco-Challenge-Sabah 2000 athletes, Malaysian Borneo, August 21–September 14, 2000.

On univariate analysis, statistically significant risk factors for illness included kayaking, swimming in the Segama River, swallowing water from the Segama River, and spelunking (Table 2). By multivariate stepwise logistic regression, only swimming in the Segama River was independently associated with illness. Adjusting for the other variables did not alter the relative risk. The attributable risk of river swimming was 36%.

**Table 2:** Risk factors for developing illness, univariate analysis^a^

Exposure	% Ill (n=80)	% Not ill (n=109)	RR	95% CI	p value
Swimming in Segama River	57	28	2.0	1.3 to 3.1	0.002
Swallowing river water	45	21	1.8	1.2 to 2.9	0.008
Kayaking	95	78	3.3	1.2 to 9.0	0.02
Spelunking	87	68	2.2	1.1 to 4.2	0.02

^a^RR, relative risk; CI, confidence interval.

Seventy-six teams, consisting of 4 members per team, competed in Eco-Challenge; at least one member of 62 (82%) of the teams was administered the questionnaire. Of the 51 teams with at least 2 members interviewed, illness and team membership were not significantly associated (data not shown).

Of the 189 athletes contacted, 20 (11%) reported taking doxycycline for prophylaxis of either malaria or leptospirosis. Seventeen of these athletes reported taking a daily dose of doxycycline (100 mg by mouth) throughout the duration of the race; the other three took daily doxycycline, 100 mg by mouth, sporadically throughout the race. Illness developed in 4 (20%) of the 20 athletes who reported taking doxycycline. When the attack rate of those taking doxycycline was compared with the attack rate among those not reporting doxycycline prophylaxis, and adjusted for river swimming, doxycycline usage was protective, although not significantly (relative risk [RR]=0.4, 95% CI=0.1 to 1.1, p=0.1). The preventive efficacy attributable to any doxycycline usage during the race was 55% (95% CI=–0.05% to 95%).

### Laboratory Investigation

Clinical symptoms of the athletes and the history of extensive water exposure led to the early consideration of leptospirosis as a diagnosis. In the first few days of the investigation, serum specimens obtained from two of the hospitalized athletes in California were tested at CDC for anti-leptospiral IgM by dot-ELISA dipstick rapid assay; one athlete had a positive test result on a serum specimen obtained 4 days after onset of fever. The second athlete had a negative test result for anti-leptospiral IgM on a serum specimen obtained 4 days after onset of fever but a positive test result on a serum sample obtained 8 days after fever onset. Based on these early results, further diagnostic testing was directed toward leptospirosis.

Of the 80 athletes who met the case definition for illness, serum was collected from 38 (48%). The median duration between onset of fever and the drawing of serum from athletes was 10 days (range 3–34). Of these 38 samples, 26 (68%) tested positive by the dot-ELISA dipstick, the IgM ELISA, or both. No differences in symptoms were found between antibody-positive and antibody-negative patients. Nine additional serum samples were obtained from ill athletes who did not meet the clinical case definition; none of these samples tested positive by rapid assay. Of the 26 samples testing positive by one or both rapid assays, 20 (77%) were subsequently determined to be positive for anti-leptospiral antibodies by MAT. The serogroup associated with the strongest MAT immunoreactivity was Australis.

One culture-confirmed isolate was obtained from an athlete in California. Based on its *rrs* and *secY* gene sequences, this isolate was determined to be most closely related to *Leptospira weilii*, a species found exclusively in Southeast Asia. Serologic analysis showed that the isolate was most likely a new serovar of the Hebdomadis group (*8*).

Eighteen samples that tested negative for leptospiral antibodies by rapid assay and MAT also tested negative for dengue, typhoid, and scrub typhus antibodies, illnesses that are clinically similar to leptospirosis. These samples included 12 from athletes who met the case definition and 6 from athletes who did not meet the definition. These 18 samples also tested negative for antibodies to Chikungunya, Powassan/tick-borne encephalitis, Sin Nombre virus, Japanese encephalitis, and Snowshoe Hare virus.

## Discussion

This outbreak was the first recognized international leptospirosis outbreak associated with the increasingly popular activity of adventure travel. Data from this outbreak investigation suggest that doxycycline may be effective as prophylaxis for leptospirosis in persons with identifiable, short-term exposure to high-risk activities and environments. Preexposure chemoprophylaxis could be increasingly important as more people engage in adventure travel and eco-tourism. In addition, physicians treating returning travelers should consider diseases such as leptospirosis in patients with a history of water exposures. Newer, rapid diagnostic assays may assist physicians in earlier and more accurate diagnosis and, therefore, earlier treatment.

Leptospirosis is a zoonotic disease of worldwide distribution that causes an acute febrile illness; the incubation period is usually 5–14 days but ranges from 2 to 30 days (9). The disease is associated with exposure to water or soil that has been contaminated by a variety of wild and domestic animals, which serve as reservoirs for leptospires and transmit infection by shedding the organisms in their urine (*1*). The illness is protean. It may be characterized by abrupt onset of fever, chills, myalgias, and headache, and may also include conjunctival suffusion, abdominal pain, vomiting, diarrhea, and skin rashes. Severe leptospirosis may include aseptic meningitis, jaundice, renal failure and hemorrhage; the more severe syndrome characterized by fever, meningismus, and renal and hepatic failure is referred to as Weil’s disease. Mild infections can be treated with oral tetracyclines; more severe infections generally require intravenous penicillin (*9*).

Among the Eco-Challenge athletes, symptoms and exposure history suggested a diagnosis of leptospirosis; this diagnosis was supported early in the investigation by laboratory testing. Our investigation showed a high attack rate in this cohort of athletes (nearly 50% in surveyed athletes). Illness was also severe, with a hospitalization rate of 36% for young, otherwise exceptionally healthy endurance athletes. On September 15, 2000, on the basis of this high attack rate and high proportion of hospitalizations, CDC recommended that all ill athletes be treated with empiric doxycycline and that asymptomatic athletes discuss the possible merits of a single dose of doxycycline with their physicians (*10*). In addition, asymptomatic athletes were advised to seek medical attention if signs and symptoms consistent with leptospirosis developed.

Although infection or coinfection with other pathogens in ill athletes remains a possibility, testing for antibodies to alternative pathogens commonly causing febrile disease in the tropics was negative in the serum samples tested; most ill athletes were likely infected with leptospires. The known epidemiology of leptospirosis and the epidemiologic data gathered from this investigation suggested a point source for the outbreak associated with exposure to water from the Segama River. The attributable risk of swimming in the river was 36%; those athletes whose illness could not be accounted for by swimming in the river were likely exposed to contaminated water or soil during some other race activity, such as kayaking, trekking, or contact with mud along the river banks. While our study did not find a significant association between swallowing river water and infection, a gastrointestinal route of infection after swallowing contaminated water has also been suggested (*11*).

Although previously reported in Malaysian Borneo, leptospirosis was not recognized as a cause of a substantial level of illness in Borneo at the time of Eco-Challenge. The known epidemiology and pathophysiology of leptospires suggest that the high attack rate among the athletes was multifactorial. Contact with water from the Segama River was preceded by prolonged treks through jungle vegetation, and all surveyed athletes reported cuts and abrasions (data not shown) that may have predisposed them to a larger inoculum of organisms (*12*). Another possible factor in the high attack rate may have been rainfall before the race. During Eco-Challenge, rainfall throughout much of the race was heavy, and in the 3 months preceding the event, regional monthly rainfall totals were approximately 250 mm greater than the average for the previous 3 years (National Oceanic and Atmospheric Administration, pers. comm.). Flooding, which has been associated with previous leptospirosis outbreaks, elevates the water table, saturating the soil with leptospires, preventing evaporation of contaminated animal urine, and potentially promoting the survival of leptospires in surface waters (*13*–*16*).

Leptospirosis is a relatively common zoonotic disease in the tropics (*9*); however, it is frequently underdiagnosed because of the nonspecific symptoms associated with infection and the difficulty confirming a diagnosis. MAT is considered the standard diagnostic criterion, but it is technically difficult, not widely available, and involves the use of live leptospires, which presents a hazard to laboratory personnel. A simple, accurate, rapid, and widely available assay for detecting leptospires is needed. In this study, initial screening for leptospires was performed with the Dip-S-Ticks immunoassay, an enzyme-linked dot immunoassay for detecting IgM antibodies; recent evaluations indicate an overall case sensitivity of 98% and specificity of 91% (*17*,*18*). The assay has benefits over previous methods of *Leptospira* serodiagnosis because of its ease of use and accessibility, as well as the rapidity of diagnosis; the Dip-S-Ticks assay also has greater sensitivity early in infection than other assays. Three Eco-Challenge athletes tested positive by Dip-S-Ticks but negative by IgM ELISA (data not shown). Detection of an early nascent immune response remains difficult; this low early sensitivity may partially explain the 50% seronegativity rate in surveyed athletes, given the 10-day median duration between onset of fever and drawing of serum. The inability to obtain MAT confirmation in all specimens that tested positive by rapid assay may similarly be explained by the relatively short interval between onset of fever and the drawing of serum. While MAT reactivity can be seen several days after infection, peak titers are frequently obtained in 2 to 3 weeks; sometimes, however, antibodies do not appear until 3–4 weeks after infection (*1*). Alternatively, serovars responsible for infection may not have been included in the testing battery used for the serum. The athletes did engage in activities that may have led to multiple exposure risks, and infection with other organisms might have accounted for a proportion of those who tested seronegative for leptospires. We tested some samples for pathogens that also cause acute febrile illness in the tropics; none was detected. However, infection with other organisms remains a possibility.

Due in part to the antigenic heterogeneity among the many different serovars of leptospires, a universally effective leptospirosis vaccine for humans has not been developed; in addition, the duration of immunity with current serovar-specific leptospiral vaccines appears to be short-lived (*19*). Prevention strategies for leptospirosis have traditionally relied on protective barriers such as wearing rubber boots and gloves and avoiding high-risk areas. However, several studies of persons from areas endemic for leptospirosis and of military recruits with no known prior exposure to leptospires have shown that doxycycline given before or shortly after exposure can reduce illness and death caused by leptospirosis (*2*–*4*). In our study, athletes who took doxycycline for malaria prophylaxis were less likely to become ill; although this finding did not reach statistical significance, we may not have had sufficient data to detect significance. Persons who travel to areas where leptospirosis is endemic or epidemic and who participate in high-risk exposure activities involving prolonged water exposure may be at increased risk for leptospirosis (*11*,*14*,*20*,*21*) and may benefit from chemoprophylaxis. Until additional efficacy data are available, persons at high risk for leptospirosis should consider chemoprophylaxis with doxycycline, 200 mg by mouth, beginning 1–2 days before exposure and continuing for the duration of suspected exposure. The role of other antimicrobial agents is not clear. Further randomized, controlled studies assessing the utility and efficacy of doxycycline and other antimicrobial agents for the prophylaxis of leptospirosis are needed.

More people are participating in exotic travel, adventure sports, and eco-tourism, and as a result, the potential for contact with pathogens less common in the temperate, industrialized world, such as *Leptospira,* is likely to increase. Outbreaks of leptospirosis have previously been documented in athletes participating in such activities as kayaking (*14*), swimming (*20*), and triathlons (*11*). Recently, leptospirosis in endurance athletes has been the subject of several reports (*22*–*24*). In addition, previous outbreaks of infectious disease attributable to other unusual pathogens have been documented in adventure travelers (*25*,*26*). Since leptospires are a common cause of febrile illness in the tropics, physicians should evaluate travelers with febrile illness returning from these areas for this and other emerging infections.
